# The Assessment of Diabetic Ketoacidosis Awareness Among Diabetic Patients and Their Caregivers in Makkah, Saudi Arabia: A Cross-Sectional Study

**DOI:** 10.7759/cureus.60336

**Published:** 2024-05-15

**Authors:** Abdulaziz A Alsaedi, Mohammed A Alsaedi, Abdullah S Eterji, Ameen A Alshenqity, Mahmoud A Alshenqity, Raghad A Alsaedi, Ruba A Alsaedi, Ziyad A Alsaedi, Bassam K Alsulami, Mokhtar M Shatla

**Affiliations:** 1 Medicine, Umm Al-Qura University, Makkah, SAU; 2 College of Medicine, Ibn Sina National College for Medical Studies, Jeddah, SAU; 3 College of Medicine, Umm Al-Qura University, Makkah, SAU; 4 Internal Medicine, Hera General Hospital, Makkah, SAU; 5 Family Medicine, Umm Al-Qura University, Makkah, SAU

**Keywords:** saudi, diabetic, caregivers, awareness, dka

## Abstract

Introduction: Diabetic ketoacidosis (DKA) is a life-threatening complication of diabetes mellitus (DM). It occurs due to a decrease in the level of insulin and an increase in the level of glucose in the blood, which makes cells unable to use glucose as an energy source and begin to break fat into ketones; an overload of ketones levels in the blood can lead to DKA. The aim of the study is to assess awareness of DKA among diabetic patients and their caregivers in Makkah City, Saudi Arabia.

Methods: This study is a cross-sectional study collected through an online questionnaire of diabetic patients and their caregivers in Makkah City. A self-reported questionnaire designed in Arabic and English through the use of Google Forms, it distributed electronically via social media to the target population with the objectives of the survey.

Results: A total of 400 participants were included, 73% of them were males, while 27% were females. A 9% of the participants have been diagnosed with DKA. A high awareness level about DKA was found in 32.5% of participants, while 67.5% had a poor awareness level. Factors associated with a high level of awareness towards DKA are young age, being single, students and having a previous DKA diagnosis.

Conclusion: Regarding our participants who have poor awareness of DKA, further education for diabetic patients and their caregivers about DKA is crucial to prevent life-threatening complications, and improve quality of life for these patients.

## Introduction

A collection of metabolic conditions known as diabetes mellitus (DM) is characterized by high blood glucose levels due to decreased insulin circulation in the body, insulin resistance, or increased synthesis of counter-regulatory hormones [1]. Globally, Saudi Arabia ranks seventh in the incidence of DM. One of the complications of DM is diabetic ketoacidosis (DKA); in Saudi Arabia, the frequency of DKA at the presentation of type 1 DM (T1DM) is very high. DKA is a life-threatening complication of diabetes and needs urgent medical attention [2]. The incidence of mortality may range from 4% to 40% in developing countries [3,4].

DKA is usually associated with T1DM, but it also can affect patients with type 2 DM (T2DM) [5]. Several papers have been done to study DKA in diabetic patients among different age groups with different methods in different countries. For example, a study among diabetic patients in Riyadh to measure their awareness of DKA showed that 38.67% have poor awareness of complications, and 67.34% have poor knowledge about management [6]. In another study, to assess the level of awareness among diabetic patients on how to avoid DKA in India, it was revealed that 80% have a poor level of knowledge [7].

According to the International Diabetes Federation (IDF), Saudi Arabia is ranked in the top 10 countries with the highest expected prevalence of diabetes in 2011 (16.2%) and 2030 (20.8%) [8]. Additionally, DKA is one of the most acute and serious complications of DM, for which treatment in many cases should be given in an intensive care unit [9]. Therefore, educating diabetic patients and their caregivers about DKA is crucial to prevent this life-threatening complication and improve the quality of life for these patients. The aim of the study is to assess awareness of DKA among diabetic patients and their caregivers in Makkah City, Saudi Arabia.

## Materials and methods

Study design and sample

A cross-sectional study was conducted from January to March 2023 among the general population of Makkah City, Saudi Arabia. Data were collected through a self-reported questionnaire using Google Forms. A self-reported questionnaire was produced using Google Forms and distributed to respondents. Through social media, such as WhatsApp groups, Twitter, and Telegram. The sample size was calculated by using the Calculator of OpenEpi (Centers for Disease Control and Prevention, Atlanta, USA). The sample size was calculated to be 384 participants. The confidence interval was 95% with considering p-value less than 0.05 to be statistically significant. We collected a total of 400 responses from the 500 questionnaires distributed which is more than the required number of participants.

Measurement tool and data analysis

Items of the self-reported questionnaire developed by authors according to a literature review. The questionnaire was conducted in Arabic and English. The study's objective and setting were explained in detail to responders on the first page of the online form. Respondents were informed that they could withdraw at any moment for any reason. The collected data would remain confidential. The inclusion criteria for this study were 15 years old and above, living in Makkah, type 1 or type 2 diabetic patient, Saudi nationality and non-Saudi nationality, and both genders included in this study. The prediabetic patient was excluded from this study. Participants were asked to fill out the questionnaire only after they had read its contents and agreed to participate in the study. Before starting the questionnaire, online informed consent was acquired.

The questionnaire consisted of three primary sections. The first section gathered sociodemographic information from respondents including age, gender, nationality, marital status, education level, employment, and income level. The second section assessed participants' history of DM and DKA. The final section assessed DKA including general awareness, causes and risk factors, signs and symptoms, and early management.

Data analysis

Data was collected through Excel sheets (Microsoft® Corp., Redmond, WA, USA) and enrolled into Statistical Package for the Social Sciences (IBM SPSS Statistics for Windows, IBM Corp., Version 21, Armonk, NY). A descriptive study was done by using frequency and percentage to summarize the data. The overall awareness level regarding DKA was graphed after assessing and summing up discrete scores for different correct awareness items. Each correct answer was given one point. The overall awareness score was categorized as a poor level if the participants' score was less than 60% of the overall score, and a good level of awareness was considered if the participant's score was 60% or more of the overall score.

## Results

A total of 400 diabetic patients and their caregivers were included. Participants' ages ranged from 15 to 64 years. The total number of participants who completed the questionnaire was 400; the total number of males was 292 (73%), while the number of females was 108 (27%). The majority of the sample (50.8%) was between the ages of 15-24. The major nationality at this was Saudi (95.3%).

Regarding educational level, 233 (58.3%) had a university degree or diploma degree and 147 (36.8%) had a secondary level of education or less, and 20 (5%) of participants had a post-graduate degree. A total of 238 (59.5%) were single and 154 (38.5%) were married. Participants were grouped by monthly household income, ranging between 3000 and 7000 SR was reported among 215 (53.8%) participants, 134 (33.5%) were employed, 195 (48.8%) were students and 71 (17.8%) were unemployed (Table 1).

**Table 1:** Personal characteristics of diabetic patients and their caregivers in Makkah, Saudi Arabia

Personal data	No	%
Age in years		
15-24	203	50.8%
25-34	54	13.5%
35-44	53	13.3%
45-54	52	13.0%
55+	38	9.5%
Gender		
Male	292	73.0%
Female	108	27.0%
Nationality		
Saudi	381	95.3%
Non-Saudi	19	4.8%
Educational level		
High school/less	147	36.8%
University/diploma	233	58.3%
Post-graduate	20	5.0%
Marital status		
Single	238	59.5%
Married	154	38.5%
Divorced/widow	8	2.0%
Monthly income		
3000-7000 SR	215	53.8%
8000-10000 SR	51	12.8%
11000-15000 SR	61	15.3%
> 15000 SR	73	18.3%
Employment		
Unemployed	71	17.8%
Student	195	48.8%
Employed	134	33.5%

Diabetes and DKA history among study participants

A total of 83 (20.8%) were patients, while 317 (79.3%) had a family member with DM. T1DM was diagnosed among 179 (44.8%) and 221 (55.3%) were diagnosed with T2DM. Among the participants, 181 (45.3%) had diabetes for more than 10 years, followed by 133 (33%) had diabetes for one to five years, and 86 (21.5%) had diabetes for 6-10 years (Table 2).

**Table 2:** Diabetes and DKA history among study participants

Diabetes mellitus (DM) and diabetic ketoacidosis (DKA) data	No	%
Were you diagnosed with diabetes mellitus (DM)?		
Yes, diagnosed	83	20.8%
Some of my family members have diabetes	317	79.3%
Type of DM		
Type 1 DM	179	44.8%
Type 2 DM	221	55.3%
Duration of DM (years)		
1-5	133	33.3%
6-10	86	21.5%
> 10	181	45.3%
Were you diagnosed with diabetic ketoacidosis?		
Yes	36	9.0%
No	364	91.0%
Do you have good information about diabetic ketoacidosis?		
Yes	101	25.3%
No	299	74.8%
In your opinion, does a diabetic patient have low knowledge about diabetic ketoacidosis?		
Yes	251	62.8%
No	149	37.3%

Exactly 36 (9%) were diagnosed with DKA; 101 (25.3%) of participants believed they had good information about DKA and 299 (74.8%) believed they had low information about DKA. Regarding participant opinion, 251 (62.8%) think diabetic patients have low knowledge about DKA.

Awareness of DKA among diabetic patients and their caregivers

Generally, 33.8% of the study participants correctly defined DKA as a complication of diabetes and requires an urgent intervention, 38.3% knew that DKA is considered as a life-threatening condition, 43.5% knew that DKA might affect other systems in the body, like the brain and heart. On the other hand, 32.3% disagreed that DKA affects children only. Regarding causes and risk factors, 29.3% know that the main reason for DKA is forgetting to take insulin injections. Regarding causes and risk factors, 83 (20.8%) are aware that physical activity may be one of the triggers of DKA, 156 (39%) reported that the incidence of DKA is decreased when the normal level of HbA1c is maintained, 20.8% know that physical exertion could be one of the causes of DKA, and 63 (15.8%) reported that infection could be one of the causes of DKA as reported by of participants (Table 3).

**Table 3:** Awareness of diabetic ketoacidosis among diabetic patients and their caregivers in Makkah, Saudi Arabia

Domain	Items		No	%
General awareness	Diabetic ketoacidosis (DKA) is considered	An emergency event occurs as a complication of diabetes and requires an urgent intervention	135	33.8%
		Normal physiological changes in response to diabetes	18	4.5%
		A chronic complication of diabetes, which occurs over a long time and doesn’t require an urgent treatment	50	12.5%
		I don’t know	197	49.3%
	DKA is considered as a life-threatening condition	Yes	153	38.3%
		No	31	7.8%
		I don’t know	216	54.0%
	DKA may affect other systems in the body, like the brain and heart	Yes	174	43.5%
		No	17	4.3%
		I don’t know	209	52.3%
	DKA affects children only	Yes	30	7.5%
		No	129	32.3%
		I don’t know	241	60.3%
Causes and risk factors	Main reason of DKA	Forgetting to take insulin injections	117	29.3%
		Long-term diabetes	43	10.8%
		Irregular eating and exercise	43	10.8%
		I don’t know	197	49.3%
	Infection could be one of the causes of DKA	Yes	63	15.8%
		No	87	21.8%
		I don’t know	250	62.5%
	Physical exertion could be one of the causes of DKA	Yes	83	20.8%
		No	79	19.8%
		I don’t know	238	59.5%
	The incidence of DKA is decreased when the normal level of HbA1c is maintained	Yes	156	39.0%
		No	24	6.0%
		I don’t know	220	55.0%

As for signs and symptoms, 176 (44%) of the study participants know about unconsciousness, followed by fatigue and tired 169 (42.3%), nausea 168 (42%), rapid or difficulty breathing 155 (38.8%), characteristic breath smell 150 (37.5%), and stomachache 127 (31.8%). Considering early management, 156 (39%) reported calling an ambulance and taking the patient to the hospital immediately (Table 4).

**Table 4:** Awareness of diabetic ketoacidosis among diabetic patients and their caregivers in Makkah, Saudi Arabia (continued)

Domain	Items		No	%
Signs and symptoms	Fatigue and tired	Yes	169	42.3%
		No	36	9.0%
		I don’t know	195	48.8%
	Unconsciousness	Yes	176	44.0%
		No	23	5.8%
		I don’t know	201	50.3%
	Nausea	Yes	168	42.0%
		No	29	7.3%
		I don’t know	203	50.8%
	Stomachache	Yes	127	31.8%
		No	46	11.5%
		I don’t know	227	56.8%
	Rapid or difficulty breathing	Yes	155	38.8%
		No	26	6.5%
		I don’t know	219	54.8%
	characteristic breath smell	Yes	150	37.5%
		No	23	5.8%
		I don’t know	227	56.8%
Early management	Urgent action if diabetic patient suffers from diabetic ketoacidosis (DKA) is	Call an ambulance, and take patient to hospital immediately	156	39.0%
		Give the patient oral sugar, and wait until the patient improves	36	9.0%
		Give the patient water, and wait until the patient improves)	24	6.0%
		I don’t know	184	46.0%

Overall awareness level of DKA among diabetic patients and their caregivers

**Figure 1:**
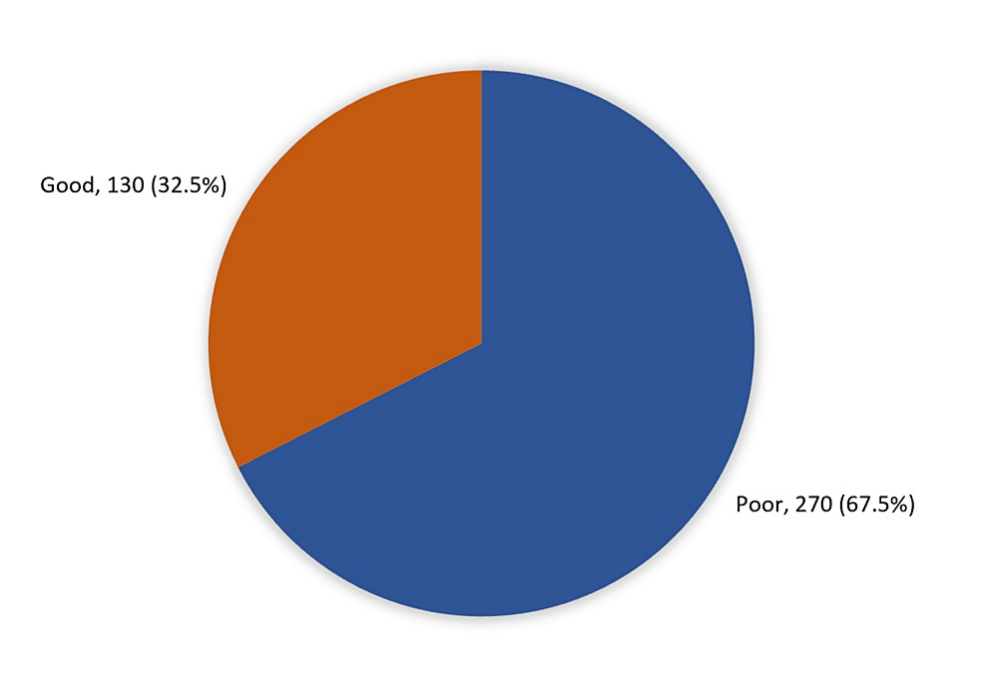
Overall awareness level of diabetic ketoacidosis among diabetic patients and their caregivers in Makkah, Saudi Arabia

Figure 1 shows that 130 (32.5%) of participants had a high awareness level about DKA while 270 (67.5%) had a poor awareness level.

Factors associated with diabetic patients and caregivers' awareness of DKA

The participants' group aged 25-34 years had an overall good awareness of DKA versus other aged groups with a statistically significant difference (P=.013). Also, the good overall awareness level among the marital status of participants showed a significant difference (P=.001). Overall, a good awareness level about participant employment had a significant difference (P=.003). Those who were diagnosed with DKA had a higher overall awareness level than those who were not diagnosed with DKA (Table 5).

**Table 5:** Factors associated with diabetic patients and caregivers' awareness of diabetic ketoacidosis P: Pearson 𝜒2 test; $: Exact probability test; * P < 0.05 (significant)

Factors		Overall awareness level				p-value
		Poor		Good		
		No	%	No	%	
Age in years	15-24	127	62.6%	76	37.4%	.013*^$^
	25-34	32	59.3%	22	40.7%	
	35-44	38	71.7%	15	28.3%	
	45-54	44	84.6%	8	15.4%	
	55+	29	76.3%	9	23.7%	
Gender	Male	195	66.8%	97	33.2%	.614
	Female	75	69.4%	33	30.6%	
Nationality	Saudi	258	67.7%	123	32.3%	.679^$^
	Non-Saudi	12	63.2%	7	36.8%	
Educational level	High school/less	94	63.9%	53	36.1%	.442^$^
	University/diploma	161	69.1%	72	30.9%	
	Post-graduate	15	75.0%	5	25.0%	
Marital status	Single	143	60.1%	95	39.9%	.001*
	Married	121	78.6%	33	21.4%	
	Divorced/widow	6	75.0%	2	25.0%	
Monthly income	3000-7000 SR	141	65.6%	74	34.4%	.751
	8000-10000 SR	37	72.5%	14	27.5%	
	11000-15000 SR	43	70.5%	18	29.5%	
	>15000 SR	49	67.1%	24	32.9%	
Employment	Unemployed	56	78.9%	15	21.1%	.003*
	Student	116	59.5%	79	40.5%	
	Employed	98	73.1%	36	26.9%	
Were you diagnosed with diabetes mellitus (DM)?	Yes, diagnosed	53	63.9%	30	36.1%	.426
	Some of my family members have diabetes	217	68.5%	100	31.5%	
Type of DM	Type 1 DM	126	70.4%	53	29.6%	.267
	Type 2 DM	144	65.2%	77	34.8%	
Duration of DM (years)	1-5	90	67.7%	43	32.3%	.851
	6-10	60	69.8%	26	30.2%	
	> 10	120	66.3%	61	33.7%	
Were you diagnosed with diabetic ketoacidosis?	Yes	11	30.6%	25	69.4%	.001*
	No	259	71.2%	105	28.8%	
Do you have good information about diabetic ketoacidosis?	Yes	16	15.8%	85	84.2%	.001*
	No	254	84.9%	45	15.1%	
In your opinion, does a diabetic patient have low knowledge about diabetic ketoacidosis?	Yes	142	56.6%	109	43.4%	.001*
	No	128	85.9%	21	14.1%	

## Discussion

The current study aimed to assess awareness of DKA among diabetic patients and their caregivers. The study showed that most participants were young, patient caregivers, while nearly one-fifth were the main diabetic cases. Also, very few per cent of the patients had DKA.

Regarding participants' awareness of DKA, the study showed that only one-third of the respondents (32.5%) were knowledgeable of DKA, whilst two-thirds (67.5%) had a low level of awareness. In more detail, about one-third of the study participants correctly defined DKA, and more than one-third knew that DKA is considered a life-threatening condition. Less than half of them reported that DKA may affect other systems in the body, like the brain and heart, but only one-third disagreed that DKA affects children only.

Our result is in line with other studies (Farran et al., 2020) [6], which found that the majority of participants have poor knowledge about the DKA regarding risk factors, management and complications. Also, another study (Alanazi et al., 2018) [10] showed that more than half of the diabetic patients in Riyadh City had a low level of awareness about the risk factors of DKA. Other research in Sudan (Elhassan et al., 2022) [11] revealed that 56.9% of participants had poor knowledge about DKA and low practice scores (0-2 out of 6). Also, a study in India (Thakare et al., 2021) [7] showed that a large majority (80%) of diabetic patients lack knowledge about DKA. As for the main cause, less than one-third (29.3%) know about forgetting to take insulin injections. The most known aggravating factor was abnormal levels of HbA1c (39%). These results differ from other studies (Satti et al., 2013) [12], revealing that infection is the major cause of DKA.

Regarding signs and symptoms, the most known were unconsciousness (44%), fatigue and tiredness (42.3%), then nausea (42%). In another study (Alazzam et al., 2022) [13], the most well-known symptom of DKA was a characteristic breath smell. While another study (Satti et al., 2013) [12] showed that the most common symptom was vomiting.

Additionally, the outcome of this study regarding factors associated with participants' awareness level showed that higher awareness level was significantly associated with having a history of DKA and young age participants who were mainly students and singles. While another study (Farran et al., 2020) [6] revealed a relationship between the level of awareness regarding DKA management and having a first-degree relative with diabetes.

However, a lower level of awareness was more common among married and unemployed participants. Also, participants who think that diabetic patient has high knowledge about DKA are significantly associated with low levels of awareness. Although, these results could be different from other studies, like the study from Hamed et al. (2021) [14]. These differences could be attributed to different samples, times and socio-economic status.

This lack of awareness can lead to significant financial and health burdens for patients and their families, as well as the country and government. Therefore, educating diabetic patients and their caregivers about DKA is crucial to prevent life-threatening complications, improve the quality of life for these patients, and enhance the health and economic system.

We recommend the Ministry of Health take a step for inverse education about diabetes and its complications, especially DKA.

Limitations

The study was done only in Makkah city, which is why it can't be generalized. Further investigations need to be done.

## Conclusions

In conclusion, the awareness level of our participants toward DKA was poor. However, a history of having DKA, a young age, being single, and being a student are associated with a higher level of awareness. Collaborative efforts and further education about DKA need to be enhanced among diabetic patients and their caregivers to prevent life-threatening complications, improve the quality of life for patients, and enhance the health and economic system. Also, further research needs to be done about diabetes and DKA to determine the level of awareness of diabetic patients and their caregivers in our area.
